# Characterization of Spatial Differences in Two Misfolded Proteins During Aggresome Formation

**DOI:** 10.17912/micropub.biology.001312

**Published:** 2024-10-21

**Authors:** Jordan N. Goldy, Robert T. Youker

**Affiliations:** 1 Department of Biology, Emory University, Atlanta, Georgia, United States; 2 Department of Biology , Western Carolina University, Cullowhee, North Carolina, United States

## Abstract

Cells have evolved an elaborate network of folding and degradation pathways to maintain the native state of proteins. If these pathways are disrupted (e.g., mutation) or their capacity is exceeded then protein aggregates form in cells. Cells sequester these aggregated proteins into aggresome or aggresome-like bodies as a protective mechanism. In this study, we co-expressed two model misfolded proteins in HEK293 cells and measured aggresome formation upon proteasomal inhibition. We observed spatial differences in early time points of aggresome formation upon co-expression of the misfolded proteins compared to individual expression in cells.

**Figure 1. Aggresome Formation upon Co-expression of GFP-250 and mCherry-cBSA in HEK293 cells:**
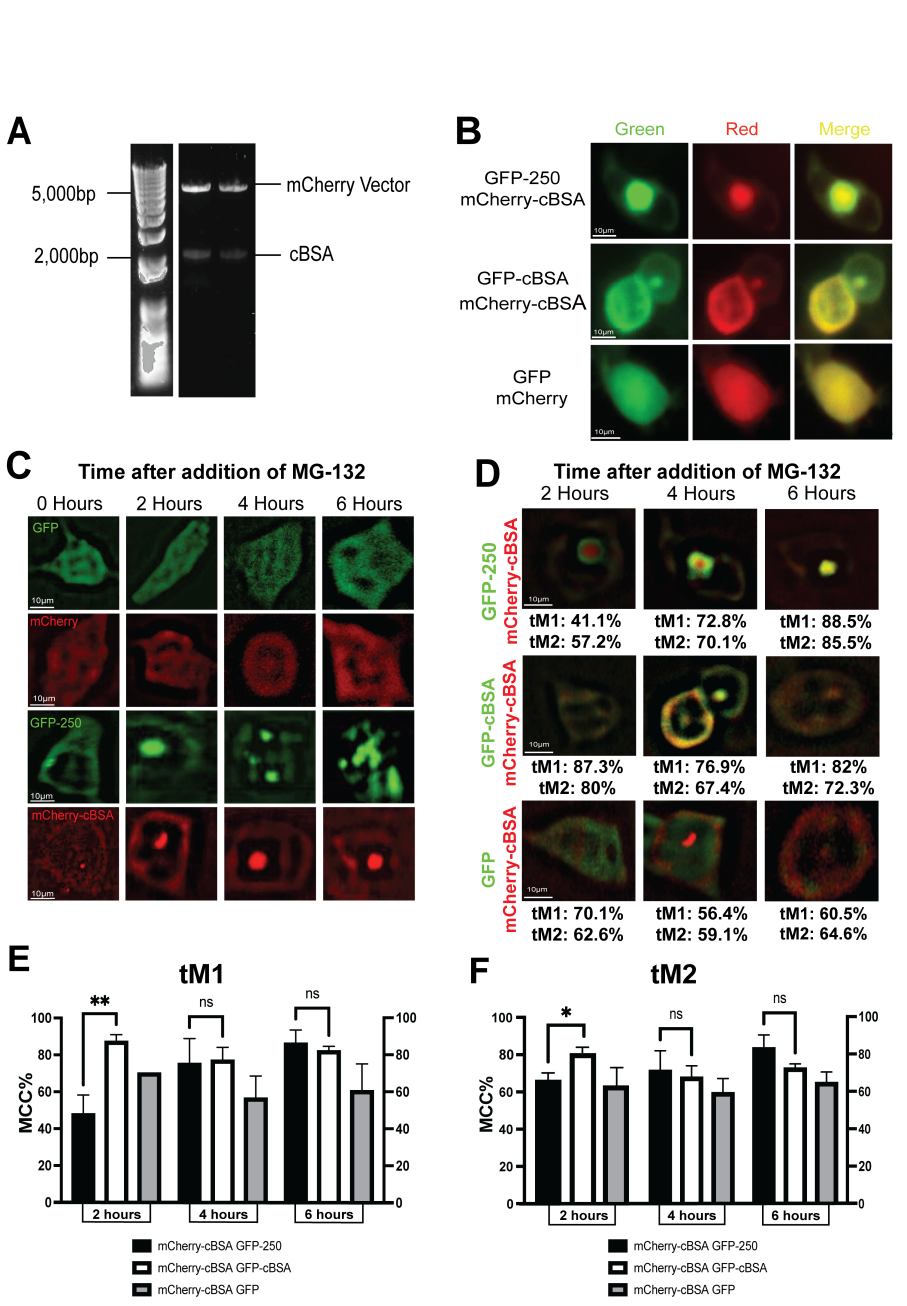
**A)** DNA Electrophoresis Gel of the mCherry-cBSA candidate plasmid clones. **B)** HEK293 cells expressing GFP + mCherry, GFP-250 + mCherry-cBSA and GFP-cBSA + mCherry-cBSA incubated with MG-132 for 4hrs. **C)** MG-132 Time course of aggresome formation. HEK293 Cells expressing GFP, mCherry, GFP-250 and mCherry-cBSA, Images deconvolved using Iterative Deconvolve 3D for ImageJ **D)** Merged Deconvolved Channels with GFP and mCherry tagged proteins with corresponding MCC colocalization percentage. tM1 represents the percentage of mCherry tagged protein colocalized with the GFP tagged protein. tM2 represents the percentage of GFP tagged protein colocalized with the mCherry tagged protein. **E)** Quantification of tM1 values (5 cells per condition/time point, 45 cells in total, N=2) **F)** Quantification of tM2 values (5 cells per condition/time point, 45 cells in total, N=2). *, p < 0.05, **, p < 0.01.

## Description

Proteins perform a plethora of functions in cells from catalysis and signal transduction to structural support. The cellular pathways that control the biogenesis, folding, transport, and degradation of proteins are called the proteostasis network (PN) (Balch et al., 2008; Hartl et al., 2011; Morimoto, 2020; Powers et al., 2009) . Disruption of one or more of the PN pathways can lead to protein misfolding causing loss of function and in many cases human disease (Balch et al., 2008; Morimoto, 2020; Powers et al., 2009) . Cells can remove misfolded proteins through multiple mechanisms, such as the ubiquitin-proteasome pathway and autophagy (Dikic, 2017; Varshavsky, 2017) . If the ubiquitin-proteasome degradation pathway and/or the protein folding capacity of the cell are overwhelmed, then protein aggregates form. Protein aggregates are assemblies of misfolded polypeptides that often become insoluble and can trigger cellular stress responses (Hetz et al., 2020; Land, 2018) . Often, these insoluble protein aggregates are sequestered by the cell in a controlled and regulated manner forming protein-dense cytoplasmic bodies (H. E. Johnston & Samant, 2021). This cellular strategy to spatially segregate misfolded/damaged proteins to prevent cellular disfunction is conserved from bacteria to humans (S. B. M. Miller et al., 2015). Originally aggresomes were characterized as perinuclear bodies surrounded by vimentin, and containing ubiquitinated misfolded proteins (J. A. Johnston et al., 1998). Many cellular structures composed of aggregated proteins (i.e., aggresome-like structures) have since been described and were given different names based on shape, composition, and/or location in the cell (García-Mata et al., 1999; Kaganovich et al., 2008; S. B. Miller et al., 2015; Ogrodnik et al., 2014; Specht et al., 2011; Spokoini et al., 2012; Szeto et al., 2006; Weisberg et al., 2012; Zhou et al., 2011) .

Two model misfolded proteins used to investigate the mechanism of aggresome formation are a chimeric protein composed of a fragment of p115 fused to GFP at its COOH terminus called GFP-250 and a cytosolic version of bovine serum albumin called cBSA that misfolds in the reducing environment of the cytosol (García-Mata et al., 1999; Qian et al., 2006; Zhang & Qian, 2011) . Previous studies that investigated cBSA and GFP-250 aggresome formation were performed in cells expressing each misfolded protein singly (García-Mata et al., 1999; Zhang & Qian, 2011) . However, it is known that co-expression of different types of misfolded proteins can lead to juxta-nuclear versus cytoplasmic peripheral aggregated bodies (Kaganovich et al., 2008; S. B. M. Miller et al., 2015) . For example, the amyloidogenic protein Huntingtin (Htt) forms insoluble peripheral deposits compared to the soluble aggregated protein von Hippel-Lindau (VHL) that accumulates next to the nucleus (Kaganovich et al., 2008) . We hypothesized that co-expression of GFP-250 and cBSA would lead to the formation of a single perinuclear aggresome given the biochemical similarities of the misfolded proteins. To test this hypothesis, we co-expressed GFP-250 and mCherry-cBSA in HEK293 cells and measured the colocalization of the protein aggregates during a time course experiment.

Initial experimental steps included digestion of cBSA-GFP and mCherry plasmids followed by ligation of cBSA into the mCherry plasmid to produce mCherry-cBSA. The parent plasmids use to produce the mCherry-cBSA were previously sequenced by researchers thus confirmation of the proper ligation of mCherry-cBSA, which was used in the following experiments, was determined via DNA gel electrophoresis **( Fig. 1 a).** The GFP-250 plasmid and the mCherry-cBSA plasmid were co-expressed together, or individually in Human Embryonic Kidney 293 (HEK293) cells and the proteasome inhibitor MG-132 was added to initiate aggresome formation. Live cell imaging was performed on cells prior to MG-132 exposure and then at two, four, and six hours after exposure **( Fig. 1 b, 1c, 1d).** Images were processed using a deconvolution technique to improve the contrast and resolution of images through reduction of out of focus light **( Fig. 1 c).**

The Costes Method for background correction was combined with Manders’ Colocalization Coefficient (MCC) to determine colocalization percentages (tM1 and tM2 values). The tM1 gives the percentage of the red channel (mCherry) that is colocalized with the green channel (GFP). tM2 gives the percentage of the green channel (GFP) that is colocalized with the red channel (mCherry). **Figure 1 d** shows the tM1 and tM2 values for images from the different conditions for the two-, four- and six-hour time points. Cells expressing both GFP-250 and mCherry-cBSA show a significant decrease in colocalization percentage at early time points compared to cells expressing both mCherry-cBSA and GFP-cBSA. Colocalization percentages for cells expressing both mCherry-cBSA and GFP-cBSA stay consistent during all time points. For mCherry-cBSA + GFP the colocalization percentage decreases over time as expected due to the change of mcherry-cBSA staining from being predominantly cytosolic to residing mostly in a perinuclear aggresome. For each condition 15 cells were analyzed (45 cells in total) and the values for tM1 and tM2 are averaged from two experiments **( Fig. 1 e, 1f)** .

The GFP-250 and mCherry-cBSA proteins were co-expressed in cells to determine if the proteins have the same dynamics of aggresome formation upon proteasomal inhibition. In the early time points, the protein aggresomes were separate with colocalization of approximately 50% for tM1 and 65% for tM2 which was significantly lower than the controls cells expressing cBSA tagged with mCherry and GFP. Later time points showed a higher colocalization 85-88% like control cells. Cells transfected with mCherry-cBSA and GFP-cBSA showed a co-expression value that ranged from 78 to 85% during the timecourse. Since the same protein (cBSA) was used for the mCherry and GFP tags it was predicted that the colocalization percentages for this condition would be high in the beginning time points and stay constant throughout the time course ( **Fig. 1 d** ). Cells with mCherry-cBSA and GFP displayed a higher colocalization at early time points which then decreased as the mCherry-cBSA formed aggresomes. For the two-hour time point an aggresome for the mCherry-cBSA was just beginning to form which led to the colocalization percentage to be its highest. The aggresomes for mCherry-cBSA grew larger thus decreasing the colocalization percentages between mCherry-cBSA and GFP. The mCherry-cBSA appears to condense into a peri-nuclear aggregate earlier than GFP-250 and then eventually both aggregates occupy the same spatial location in the cell at later time points (two- and four-hour). This result does not fully support our original hypothesis that the dynamics of aggregation are identical for these two proteins upon co-expression in the same cell. Overall, these data suggest that GFP-250 and mCherry-cBSA proteins may have different mechanisms of aggresome formation upon co-expression in cells. Further biochemical and cellular studies will be needed to ascertain the cause for this difference in aggresomal dynamics.

## Methods

*Cloning:* A double digestion of cBSA-GFP and mCherry was carried out with BamHI-HF and SacI-HF at 37 ^o^ C for 1 hour. The digested mCherry plasmid was treated with shrimp alkaline phosphatase (SAP) before ligation with the cBSA gene using T4 DNA ligase. Successful ligation was confirmed via DNA electrophoresis gel.

*Cell Culture:* HEK293 cells were cultured from frozen stock in Dulbecco’s Modified Eagle Medium (DMEM, Invitrogen) and supplemented with 10% (v/v) Fetal Bovine Serum. Cells were incubated at 37°C with 5% CO2 and 95% humidity. Cells were routinely passaged twice a week when cells reached ~80% confluency and low passage numbers were used for experiments (3-5 passages).

*Transfection* : Day 1: The day before transfection, 250,000 HEK293 cells were plated in each well of a six-well plate. Media used was DMEM supplemented with 10% FBS with 2 ml of media per well. Day 2: 250ng of each plasmid was transfected into cells using Continuum transfection reagent (GeminiBio) according to manufacturer’s instructions. Day 3: 42 mM MG-132 was dilution 1:5 in DMSO and 4.75 μl of MG-132 was added to each well of a six-well plate for a final concentration of ~20 μM.

*Imaging* : Live cell imaging was performed using the Evos FL Auto microscope with a 40x 0.65NA objective. GFP, GFP-250 and GFP-cBSA were imaged using GFP light-cube with an excitation of 470/22nm and emission of 510/42nm. mCherry and mCherry-cBSA were imaged using TexasRed light-cube with an excitation of 585/29nm and emission of 624/40nm. For the TexasRed channel the following settings were used: light=58, exposure=575 msec and gain=12. For the GFP channel the following settings were used: light=19, exposure=193 msec and gain=9. Images were captured 2 hours, 4 hours, and 6 hours after addition of MG132.

*Analyses* : Each image captured by the microscope was deconvolved using the plugin Iterative Deconvolve 3D for ImageJ using default settings and 10 iterations (Dougherty, 2005) . After deconvolution the percent colocalization of the images were calculated using the Costes method for background determination followed by Manders' Colocalization Coefficient (MCC) calculation in ImageJ (Schindelin et al., 2012) . Non-transfected cell images are not required for the Costes method as this is a robust, reproducible and non-user bias method for assessing background signal intensity (Dunn et al., 2011) . The value for tM1 is the fraction of the red channel that is colocalized with the green channel above the background threshold. The value for tM2 is the fraction of the green channel above threshold that is colocalized with the red channel (Dunn et al., 2011) . For each time point the average for the tM1 and tM2 percentages were calculated. Cell counts were averaged from two representative experiments. Students t-test was performed to determine statistical significance between sample conditions using Prism 10.

## Reagents

**Table:** 

**Plasmid**	**Acquired From**
pEGFP-C2-p115_1-252 amino acids (GFP-250)	Dr. Elizabeth Sztul (University of Alabama at Birmingham)
pcDNA3.1(+)-mCherry (mCherry)	Dr. Jennifer Pluznick (Johns Hopkins University)
pEGFP-N1-cBSA (cBSA-GFP)	Dr. Shu-Bing Qian (Cornell University).

**Table:** 

**Enzyme**	**Available From**
BamHI-HF	NEB, catalog # R3136S
SacI-HF	NEB, catalog # R3156M
T4 DNA Ligase	NEB, catalog # M0202L
rSAP (shrimp alkaline phosphatase)	NEB, catalog # M0371S

**Table:** 

**Reagent**	**Available From**
MG-132	InvivoGen, catalog # Tlrl-mg132-2
